# Longitudinal study of long-term smoking behaviour by biomarker-supported determination of exposure to smoke

**DOI:** 10.1186/1471-2458-14-348

**Published:** 2014-04-12

**Authors:** Anthony Cunningham, Johan Sommarström, Ajit S Sisodiya, Graham Errington, Krishna Prasad

**Affiliations:** 1 Group Research and Development, British American Tobacco (Investments) Ltd, Regents Park Road, Southampton SO15 8TL, UK

**Keywords:** Longitudinal study, Biomarker of exposure, Mouth-level exposure, Smoking, Compensatory smoking behaviour

## Abstract

**Background:**

Long-term studies of smokers who switch to lower nicotine yield cigarettes have been identified by the World Health Organization Study Group TobReg and the US Food and Drug Administration as one key area where new knowledge is required to guide science based regulation. The limited number of long-term switching studies have concluded that smokers who switch to lower nicotine yield cigarettes show evidence of partial compensation. Since the European Union tobacco product directive of 2001 introduced tar and nicotine yield ceilings, there has been no long-term observational switching study. To address the limitations of previous studies where smokers were forced switched for relatively short durations, we plan to undertake a long-term study of spontaneous switching which is appropriately powered and includes non-switchers as a control group.

**Methods/design:**

Healthy adult smokers aged 21–64 years will be enrolled into this 5-year non-residential, multicentre study across 10 cities in Germany. They will be assessed at 10 timepoints with 6 month intervals during which inclusion criteria will be reassessed and spent cigarette filter tips, saliva and 24 h urine samples will be collected. These samples will be used to determine average daily cigarette consumption, estimate mouth-level exposure to tar and nicotine and measure selected biomarkers of exposure, respectively. Spontaneous changes in subjects’ preferred cigarette products and any consequent change in tar or nicotine yield will be monitored. Subjects will be required to complete questionnaires on quality of life, smoking behaviours, smoking-related sensory attributes and recent life changes.

**Discussion:**

The planned study is anticipated to contribute to understanding smokers’ behaviours and their consequent exposure to smoke constituents. It will also allow assessment of compensatory changes in their behaviour following spontaneous switching of cigarette product smoked. Data from this study are expected to provide insights into study design and conduct for non-clinical assessment of smokers’ exposure as part of post marketing surveillance programmes.

**Trial registration:**

Current Controlled Trials Database reference ISRCTN95019245.

## Background

The long-term risks associated with cigarette smoking, particularly cancer [1,2], cardiovascular disease [3] and chronic obstructive pulmonary disease [4], are well established. Few longitudinal studies, however, have assessed spontaneous changes in smoking behaviour, and especially whether a smoker’s nicotine uptake remains constant or changes over time. Studies in adult smokers have shown stable values for cigarette consumption and concentrations of carboxyhaemoglobin (a biomarker for exposure to carbon monoxide) over periods of several months when smoking the same product [5-7].

### Previous longitudinal studies of spontaneous product switching

A small number of longitudinal studies have investigated the effects of spontaneous product switching on smoking behaviour [8-12] and the extent of compensatory smoking behaviour. Compensation occurs when a smoker changes their smoking behaviour in some way, for example, puff volume, frequency or the number of cigarettes smoked, in response to smoking a cigarette of higher or lower smoke yield. Complete compensation occurs when a smoker maintains their intake following a change, no compensation results in a change that is proportional to the change in product yield, and incomplete compensation describes changes between these two extremes [13]. In a study in the USA, conducted by the American Cancer Society between 1959 and 1972 on changes in cigarette consumption in relation to changes in tar and nicotine yield, data from 28,561 male smokers were reported [8]. By the end of the study, 16,991 (59%) smokers had changed to cigarettes with lower tar and nicotine yields than at the start of the study, 8,190 (29%) had not changed, and 3,380 (12%) had changed to cigarettes with higher yields. Among smokers who had switched to cigarettes with increased tar and nicotine yields, 29% had also increased the number of cigarettes they smoked per day, while 39% had decreased the number smoked. Likewise, although 32% of smokers who had switched to lower tar and nicotine products smoked more cigarettes per day, 34% smoked fewer. The data suggest that changes in cigarette consumption were not related to smokers changing to cigarettes of higher or lower tar and nicotine yields, though the yields of tar and nicotine in the cigarettes smoked during this period would have been significantly higher than those typically seen today in most countries.

The effects of changing to lower tar yield cigarettes was investigated over a period of 13 years (1971–1984) in nearly 600 male smokers [9]. Average cigarette consumption reduced over the study period, but to a lesser extent in smokers who had changed to lower tar cigarettes than in those who had not. At the 1984 follow-up, similar amounts of urinary nicotine metabolites were excreted by smokers who had and had not changed to lower tar cigarettes at data collection, which suggests compensatory smoking behaviour following switching. In a study of the effects of spontaneous switching on nicotine and carbon monoxide exposure in 203 smokers, a reduction in cigarette consumption of 6.6 cigarettes per day was observed in smokers who changed spontaneously to lower nicotine yield cigarettes, with smaller reductions in consumption for smokers who had not changed product or who had changed to higher nicotine yield cigarettes (1.9 and 1.8 fewer cigarettes, respectively) [10]. For the smokers who changed to lower nicotine yield cigarettes, absolute plasma cotinine levels were reduced, however, plasma cotinine levels per cigarette were little altered, which suggests an increase in smoking intensity. In a longitudinal study in South Germany, 41 smokers changed their cigarette during the study, 13 of whom changed to a lower tar product [11]. The extent of the reductions in cotinine concentrations suggested 55% compensation.

In 2002–2005, Muhammad-Kah and colleagues assessed spontaneous product switching in 2,542 smokers in the USA, of whom 68 switched to higher tar products and 23 switched to lower tar products, with subsequent decreases of two cigarettes and increases of two cigarettes per day, respectively [12]. Substantial variability and insufficient power to detect significant differences in biomarkers of exposure, however, led to no definitive conclusions being reported.

Since 2004, in the European Union the maximum permitted tar and nicotine yields have been 10 mg and 1 mg per cigarette, respectively, measured using International Organization for Standardization (ISO) methods [14]. Following the introduction of these regulations, long-term changes in consumption [15], nicotine uptake, exposure to smoke toxicants and the effects of switching to cigarettes of lower yields are unclear. The World Health Organization (WHO) Study Group on Tobacco Product Regulation (TobReg) identified a number of research areas as being key to enhancing this knowledge [16], which include assessment of whether an increase or decrease in nicotine content per unit (e.g. per cigarette) would be beneficial to a population’s health and investigation of contents and design features that might reduce toxicity, consumer appeal and/or the potential for dependency. In 2009, the US Food and Drug Administration was given jurisdiction over tobacco products via the US Family Smoking Prevention and Tobacco Control Act, and in 2012 issued draft guidance on the key areas to be investigated in making marketing applications for modified-risk tobacco products, which includes the requirement for post-market surveillance studies [17]. The FDA is also considering product standards, which could involve the limiting of harmful and potentially harmful constituents and nicotine.

### Study objectives

We plan to undertake a longitudinal study to address some of the TobReg research aims [16], to provide insights into appropriate study designs for undertaking post marketing surveillance programmes. We will study the effects of spontaneous product switching over a 5 year period on the following features of smoking behaviour: average daily cigarette consumption, mouth-level exposure (MLE) to nicotine and tar [18]; uptake of nicotine, assessed by measurement of urinary and salivary biomarkers of exposure; and exposure to the smoke toxicant 4-(methylnitrosamino)-1-(3-pyridyl)-1-butanone ([NNK] a tobacco-specific nitrosamine) through the measurement of metabolites in urine. Secondary objectives are to assess any compensatory smoking behaviour and changes in quality of life and sensory perception following spontaneous product switching.

## Methods/design

### Study design

**Figure 1:**
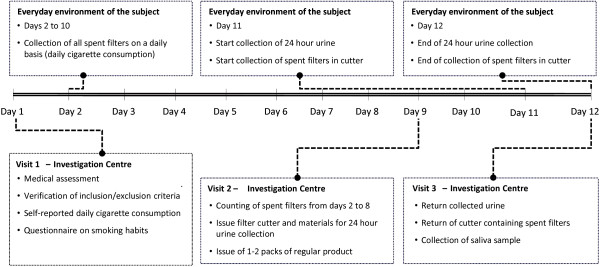
Study activities during fieldwork periods.

The study will be performed in Germany over 5 years and will involve 10 non-residential study centres. The study will comprise 10 timepoints over the 5-year period, each of 6 months’ duration. Each timepoint will include a 12-day fieldwork period consisting of three clinical visits separated by ambulatory periods (Figure 1, Table 1) and telephone based interviews between the fieldwork periods.

**Table 1:** Study activities per timepoint

	**Day 1 Visit 1**	**Days 2 to 8**	**Day 9 Visit 2**	**Day 10**	**Day 11**	**Day 12 Visit 3**	**Call 1**	**Call 2**	**Call 3**
Informed consent^a^	X								
Demographic data (height^b^ and weight)	X								
Medical history	X								
ECG^b^	X								
Spirometry (FEV_1_)	X								
Blood pressure	X								
Inclusion/exclusion criteria	X								
**Materials provided to Subject**									
Urine collection bottles			X						
Aluminium containers for butt collection	X		X						
Issue of cigarettes for 1 day			X						
Filter cutters with integrated tin			X						
Sensory questionnaire			X						
**Subject activities**									
Usual subject environment		X		X	X		X	X	X
Smoke own supply of cigarettes	X	X	X	X		X	X	X	X
Collection of cigarette butts		X	X	X					
Collection of part-filter tips					X				
Start 24 hour urine collection					X				
End 24 hour urine collection						X			
Smoke provided cigarettes					X				
Complete questionnaires	X		X		X	X			
Telephone interview				X			X	X	X
**Provided by Subject**									
Butts			X			X			
Urine						X			
Sensory questionnaire						X			
Filter tip						X			
Saliva						X			

^a^Informed Consent will be taken at visit 1, first fieldwork period only or if updated.

^b^Height and ECG will be measured at visit 1, first fieldwork period only.

The protocol and the informed consent form have been reviewed and approved by the Ethics Committee of the Bavarian State of Chamber of Physicians (Ethik-kommission der Bayerischen Landesärztekammer), Munich, Germany (Ethics Committee Number 08036) as the central voting ethics committee for the study. Approval was given by all local ethics committees relevant to the site investigators (Sächsische Landesärztekammer, Ärztekammer Nordrhein, Landesärztekammer Baden-Württemburg, Ärztekammer Hamburg, Ärztekammer Berlin). The principal investigator or designated investigators will ensure that subjects are provided with oral and written information regarding the study, associated activities and storage of data and samples. Subjects will be required to read, sign and date informed consent forms before enrolment in the study. The study will be conducted by an independent contract research organisation (CRO), Harrison Clinical Research Deutschland GmbH, Munich, Germany, in accordance with the ethics principles of the Declaration of Helsinki [19] and the International Conference on Harmonisation Guidelines for Good Clinical Practice [20]. Subjects will be compensated for the inconvenience and effort in participation in the study, at rates approved by the ethics committee of Bayerischen Landesärztekammer.

### Study population

#### Identification of subjects

Male and female adult smokers of Lucky Strike Red King Size cigarettes (LSR) (ISO yields 10 mg tar/cigarette and 0.8 mg nicotine/cigarette) will be eligible. Recruitment of subjects will be undertaken by a market research agency (MRA), Ipsos Operations GmbH, Hamburg, Germany. Potential subjects will be selected from people approached in the streets in the vicinity of shops selling tobacco products (kiosks, newsagents, grocers etc.) and those who respond to local newspaper advertisements.

#### Inclusion criteria

Subjects will be required to satisfy the following inclusion criteria: age 21–64 years at the time of signing the informed consent; female subjects must not be pregnant or lactating at the time of enrolment; be in good health, as determined by medical history, baseline 12-lead electrocardiography (ECG), lung-function tests and measurement of blood pressure; smoke eight or more LSR cigarettes per day and have done so for at least 6 months at the time of enrolment; willingness to provide written informed consent to participate in the study; and willingness to purchase cigarettes for personal use. Continued eligibility for inclusion according to these criteria will be reassessed at each timepoint during the study.

#### Exclusion criteria

Subjects who fulfil any of the following criteria will not be enrolled: having an existing chronic disease, such as asthma, cardiovascular disease, chronic obstructive pulmonary disease, or a history of lung cancer or any other tobacco-related disease, as determined by the principal investigator and/or site investigator; participation in another study of smoking habits in the previous 6 months; trying to stop smoking or considering doing so in the next 2 months; a candidate or an immediate family member working in public relations or advertising for the tobacco industry, including the sale or manufacture of tobacco goods; consumption of tobacco products other than factory-made cigarettes available on the German market (e.g. products that do not comply with the German Tobacco Ordinance Act, Tabakverordnung [TVO], smokeless tobacco products, and non-filtered products such as cigars or pipes); and candidates who in the opinion of the principal investigator and/or site investigator should not participate in the study. These exclusion criteria will be reassessed at each timepoint.

#### Subject withdrawals

After enrolment subjects are free to withdraw from the study at any time. In addition, the principal investigator and/or site investigator may decide to withdraw a subject if a protocol violation is suspected. The date and reasons for the withdrawal will be clearly stated in the subject’s case report form. No subjects will be withdrawn for not attending scheduled visits or calls during the fieldwork periods or timepoint, provided they can be contacted later and can provide a reason for non-attendance. Otherwise, they will be entered into the lost-to-follow-up process. Withdrawn subjects will not be replaced.

### Fieldwork periods

Fieldwork periods will cover the first 12 days of every timepoint (Figure 1). Subjects will visit the study centres three times during these 12 days (visits 1, 2 and 3), on days 1, 9 and 12. Day 1 of the first fieldwork visit will be used to assess the eligibility of subjects according to the inclusion and exclusion criteria and to assign each subject with a unique study code. The subjects’ identification and study codes will be checked at each visit.

During visit 1, medical history and demographic data will be updated, and blood pressure, spirometry, weight, height and ECG (latter two, first fieldwork period only) will be assessed. Subjects will be given aluminium containers for daily collection of cigarette butts from all cigarettes smoked on days 2–9 and instructed verbally how to collect them. Interviewer-led questionnaires on smoking behaviour will be completed.

During visit 2, the butts from days 2–8 will be counted to calculate average daily consumption and cigarette brands will be checked. Aluminium containers will be supplied for collection of cigarette butts on day 10, along with a filter cutter/collector, and instructions for the collection of part-filters for filter analysis from cigarettes smoked on day 11. Polyethylene containers, also for use on day 11, will be provided with verbal and written instructions for 24 h urine collection along with a report sheet on which to record any medications taken during this period. A 1-day supply of the subjects’ current cigarettes will be provided for smoking on day 11. A sensory evaluation questionnaire will be provided for self-completion during day 11.

During visit 3, the used cigarette butts collected on days 9 and 10, the 24 h urine sample and cigarette part-filter samples from day 11 and the self-completed sensory questionnaires will be collected. Saliva samples will be taken under the supervision of a study nurse, for analysis of the nicotine metabolites cotinine and *trans*-3′-hydroxycotinine (OH-cotinine). A self-reported questionnaire on recent life changes and interviewer-led questionnaires on smoking behaviour and quality of life will be completed.

Study staff will also contact participants by telephone four times during each timepoint, at approximately 2 and 4 months after the last fieldwork visit (calls 1 and 2), 1 week before the next fieldwork period (call 3) and on day 10 of fieldwork periods (call 4). During telephone contact, the following activities will take place: calls 1 and 2 will be interviews with subjects to verify they still meet the original non-medical inclusion criteria and to record which cigarette products are currently being smoked, reasons for any change in current cigarette product smoked since the previous timepoint and self-reported daily consumption; call 3 will be to remind subjects of their visit 1 appointment and determine whether they have changed their cigarette product; call 4 will be to remind subjects to collect part-filter tips and urine on day 11.

### Study procedures

#### ECG assessment

During visit 1 of the first fieldwork period only, subjects will undergo 12-lead resting ECG with a 10 s rhythm. Subjects will be supine for at least 5 min before the test. The ECG will be performed, documented and assessed by a physician. Any subject with an abnormal rhythm or trace shape on ECG will be excluded from the study and advised to consult a cardiologist. ECGs may be undertaken in other fieldwork periods if a subject reports relevant symptoms, but he or she will be advised to consult a cardiologist and a copy of any follow up report will be retained.

#### Blood pressure and pulse rate assessments

Supine blood pressure and pulse rate will be measured during visit 1 of the first fieldwork visit. Subjects will be supine for at least 5 min before assessments. During visit 1 of all subsequent fieldwork periods, blood pressure and pulse rate measurements will be taken with subjects in the sitting position. Blood pressure assessment will also be performed at other times if judged to be clinically appropriate by the study staff.

#### Spirometry

Spirometry will be used to measure forced expiratory volume in 1 s (FEV_1_) as evidence of changes in pulmonary function during the course of the study. Lung-function tests will be performed at visit 1 of all fieldwork periods. All subjects will stand during lung-function measurements and the best of three technically acceptable attempts will be taken as the true measure of lung function for each timepoint. At least one other attempt must give a result within 5% of the best value for the average to be valid. If necessary, measurements will be repeated until two attempts within 5% of each other are obtained. If a subject shows signs of fatigue, testing will be halted and will only recommence at the physician’s discretion.

#### Cigarette butt collection

In every fieldwork period, subjects will be provided with labelled aluminium containers, and instructed to collect butts from all cigarettes smoked on days 2–9 in those aluminium containers provided at visit 1, and the butts from cigarettes smoked on day 10 in the containers provided at visit 2.

During visit 2, the collected cigarette butts from days 2–8 will be counted at the study centres to calculate average daily cigarette consumption and identify the brands smoked, where possible. After counting, the butts will be discarded. Subjects who have deviated from their normal cigarette consumption (defined as an increase or decrease in consumption on ≥1 day by more than 25% of the average daily consumption from days 2–8) will complete an interviewer-led questionnaire to identify the reasons for the deviation. At visit 3, the collected butts for days 9 and 10 will be counted in the same manner.

#### Smoking and part-filter collection

On all days during the study, except day 11 of the fieldwork periods, subjects will purchase and smoke their own cigarettes. On day 9 of every fieldwork period, cigarettes that match each smoker’s current product will be provided in sufficient quantity to match average daily consumption, along with a labelled filter cutter with integrated collection tin. The subjects will be requested to smoke only these cigarettes on day 11, from the first to the final cigarette of the day, and to use no other nicotine-containing products during this 24 h period. Subjects will be instructed to smoke the provided cigarettes as usual and to cut off and collect 10 mm mouth end sections of the filter tips from every cigarette smoked that day. To minimise the possible influence of changes in smoking behaviour caused by social circumstances and environment, fieldwork periods will be scheduled to ensure that the collection of filter tips will occur only on working days. The number of filter tips in the filter cutter will be counted on day 12.

Filter tips must meet the quality guidelines of the sponsor (i.e. not crushed or containing ash and of approximate correct length). If they are not of adequate quality, subjects will collect them again on the next available working day, along with repeating the 24 h urine collection. The collected cigarette filters will be stored at room temperature pending dispatch to the sponsor for analysis of tar and nicotine content. These data will be compared with calibration curves from a range of machine smoking regimes, where the nicotine and tar yields and filter nicotine and tar contents are known and the MLE to nicotine and tar per cigarette calculated [21]. Daily MLE estimates will be calculated by multiplying the per-cigarette values by the number of cigarettes consumed on day 11 of the fieldwork periods.

#### Urine collection and processing

Urine will be collected into standard-weight polyethylene containers. The 24 h collection period will always be on working days to minimise the possible influences of social circumstances and environment on smoking behaviour. The collection period will commence after the first morning void, which will be discarded, and will continue throughout day 11 and include the first morning void on day 12. The times of the first void, first collection and last collection will be recorded by subjects on the provided report sheets. During each 24 h collection period, the containers will be stored in a cool bag with two frozen cooling elements.

Subjects will document on the provided report sheets any medications taken during the 24 h urine collection period. The bulk urine for each subject will be frozen within 15 min of receipt and transported to the CRO, where it will be stored at approximately –20°C until analysis, when it will be thawed and the weight recorded. A nominal value for specific gravity (1.018) will be used to calculate urine volume [22].

After the bulk urine has completely thawed, aliquots will be transferred into suitably labelled polypropylene tubes and stored at approximately –20°C pending dispatch for analysis by ABF GmbH, Munich, Germany, and long-term storage at Covance, Leeds, UK.

Urine samples will be analysed for the concentration of nicotine and five main metabolites (cotinine, OH-cotinine, nicotine-*N*-glucuronide, cotinine-*N*-glucuronide and *trans*-3′-hydroxycotinine-*O*-glucuronide) and the metabolites of NNK, 4-(methylnitrosamino)-1-(3-pyridyl)-1-butanol (NNAL) and NNAL-glucuronides (total NNAL). Creatinine concentration will be determined to provide an indication of urine collection compliance.

#### Saliva collection

On visit 3 of each fieldwork period, saliva samples will be collected after 15:00 h from all subjects, for measurement of the nicotine metabolites cotinine and OH-cotinine. Samples will be collected with Salivettes (Sarstedt, Nuembrecht, Germany). Subjects will be asked to place a cotton wool swab in their mouth and move the swab with a slight chewing action to allow for saturation with saliva over a 2 min period. Within 15 min of collection the samples will be frozen and stored at approximately –20°C, pending dispatch for analysis.

#### Monitoring of changes in cigarette consumption

Checks on whether each smoker has changed their main cigarette product will be carried out at calls 1, 2 and 3 of each timepoint. The products reported by the subjects during call 2 will be obtained by the sponsor for supply at the next fieldwork period. Call 3 will identify any changes in a subject’s cigarette product smoked, one week before the fieldwork period. Change to cigarettes with a lower ISO tar yield compared to those smoked at baseline (e.g. from 10 mg to 6 mg), a higher ISO tar yield (e.g. from 6 mg to 10 mg) and to a different cigarette with the same ISO tar yield (e.g. one 10 mg ISO tar product to another 10 mg ISO tar product) will be permitted. Subjects will be considered no longer eligible if they change their smoking habits in any of the following ways: change to smoking cigarettes that are not TVO compliant; change to smoking non-filtered products; change to smoking self-rolled (roll your own) cigarettes or self-filled “stix” (make your own) cigarettes, with or without filters; change to using chewing tobacco or other smokeless tobacco products; and use of nicotine-replacement therapy or decision to quit smoking.

The MRA will maintain all information regarding switches in products and smoking behaviour.

#### Completion of questionnaires

On day 1 of visit 1 of every fieldwork period, subjects will complete an interviewer-led electronic questionnaire on smoking behaviour, including the Fagerström Test for Nicotine Dependence [23]. Questionnaires about deviation from normal cigarette consumption will be completed during visit 2 of fieldwork periods, on day 9, and questionnaires on sensory attributes will be provided for self-completion at home on day 11. These will be returned on day 12 at visit 3. During visit 3, subjects will also be required to self-complete the Recent Life Changes questionnaire [24] and the interviewer-led SF-36v2 Quality of Life questionnaire [25]. The responses to the self-completed questionnaires will be entered into an electronic data capture system at the study centres by study personnel.

#### Lost to follow-up

Subjects will be permitted to miss whole timepoints provided they are contactable and can give valid reasons for doing so. Subjects who cannot be contacted during scheduled events (visits or calls) will enter the “lost-to-follow-up” process. The process starts with a telephone call, followed up by a second call two weeks later, if the subject makes no response. If there is still no response from the subject, he or she will be sent a letter by fax or e-mail if contact information is available. The final step in this process is that a registered letter will be sent to the subject. If the subject responds to any contact, the process will be stopped. Otherwise, the subject will be withdrawn from the study by the principal investigator.

### Sample size and power calculations

It is expected that the subjects will separate into three groups during the study: subjects who do not change product from the original 10 mg ISO tar product throughout the study; those who change to cigarettes with ISO tar yields of 7 mg or lower (down-switchers); and those who change to cigarettes with ISO tar yields of 8–10 mg (side-switchers). A fourth group is possible that will include subjects who down-switch but subsequently change back to cigarettes of higher tar yields (up-switchers). Subjects cannot up-switch from the original 10 mg ISO tar product due to the upper limit of 10 mg ISO tar for cigarettes sold in the EU [14]. Analysis of biomarkers of exposure in sub-populations of smokers and non-smokers in Germany indicated that differences of 20% between the subject groups for each respective biomarker would yield results significant at an alpha level of 0.05 where group sizes were greater than n = 50 [26]. Power and sample size calculations were also used to determine thresholds required to detect significant differences between subject groups. These calculations showed that to achieve a power of 80%, a sample size of 51 will be required to detect differences of 2 mg in MLE to tar between subject groups [26]. To achieve a minimum of 51 subjects in each of the three subject groups over multiple timepoints and taking account of predicted product switching rates (3.7% down-switching and 10% side-switching), and allowing for an annual reduction of 20.4% (based on a combination of subject withdrawal and attrition from the study and an annual quitting smoking rate of 12.4%), it will be necessary to recruit 1000 subjects. Based on the predicted annual reduction of 20.4%, it is predicted that 319 subjects will complete the study.

### Statistical analysis

The statistical analyses to address the primary and secondary objectives will be performed on the final per protocol population. Data will be tested for normality and homogeneity of the variance and, if appropriate, data will be transformed to try to satisfy analysis of variance (ANOVA) assumptions. If normality approximation cannot be achieved, non-parametric methods may be employed. A linear mixed model will be used to assess group changes over time for all MLE, biomarkers of exposure and consumption measurements. The model will include fixed effects due to subject group (non-switcher, down-switcher or side-switcher), timepoint, gender, subject group*timepoint interaction and age as a covariate. The interaction term will be removed from the model if not statistically significant. Data from the first timepoint will be used to account for possible subject differences at the start of the study. Repeated measures ANOVA will be performed to account for the correlation over time within each subject.

Established procedures will be used to determine the extent of any compensation effect, as a result of subjects switching products. These procedures calculate compensation indices on the basis of machine-smoked yields and exposure data [13,27].

A detailed statistical analysis plan of the methodology to be used will be prepared before the database closure.

## Discussion

The planned study is the first longitudinal study of smokers’ exposure to current cigarette products in over 20 years and has been designed to contribute to the understanding of exposure of smokers over the long-term to cigarette smoke and selected constituents and any changes in consumption. The effects of switching to cigarettes of different tar and nicotine yield have been extensively studied, yet very few studies have considered spontaneous product switching, where the smokers have chosen their new product. Understanding of exposure to the constituents of tobacco smoke after smokers change products will be increased and whether smokers make any compensatory changes to their smoking behaviour assessed. The data may also show whether changing to products with lower tar and nicotine yields would have any benefits for the smoker with respect to quality of life.

Data from this study are expected to contribute to the development and long-term assessment of tobacco products with reduced contents of harmful substances following their introduction on to the market, as part of post-marketing surveillance programmes [16,17,28,29]. In the next few years, new methods to analyse biomarkers of exposure to tobacco products are expected to be available. The use of these new methods on samples in this study could greatly increase the amount of knowledge gained. The results of this study will be published in peer-reviewed scientific journals.

## Abbreviations

ANOVA: Analysis of variance; CRO: Contract research organisation; ECG: Electrocardiography; FEV1: Forced expiratory volume in 1 s; ISRCTN: International Standard Randomised Controlled Trial Number; ISO: International Organization for Standardization; MLE: Mouth level exposure; MRA: Market research agency; NNAL: 4-(methylnitrosamino)-1-(3-pyridyl)-1-butanol; NNK: 4-(methylnitrosamino)-1-(3-pyridyl)-1-butanone; OH-cotinine: *trans*-3′-hydroxycotinine; TobReg: Tobacco Product Regulation; TVO: Tabakverordnung (German Tobacco Ordinance).

## Competing interests

AC, JS, ASS, GE and KP are current employees of British American Tobacco and the work to be funded by British American Tobacco.

## Authors’ contributions

ASS and KP developed the study concept and design. GE contributed to the study design and performed the sample size and power calculations. All authors contributed to the development of the study protocol. AC drafted the manuscript. All authors read and approved the final manuscript.

## Pre-publication history

The pre-publication history for this paper can be accessed here:

http://www.biomedcentral.com/1471-2458/14/348/prepub
